# Case Report: Bilateral Wilms tumor with TP53 mutation: a case-based review of clinical challenges

**DOI:** 10.3389/fsurg.2026.1759786

**Published:** 2026-05-21

**Authors:** Xin Zhang, Qianqian Cheng, Pinxiu Wang, Zhen Tan

**Affiliations:** 1 Department of Pediatric Hematology/Oncology, School of Medicine, Xinhua Hospital, Shanghai Jiao Tong University, Shanghai, China; 2 Pediatric Surgery, School of Medicine, Xinhua Hospital, Shanghai Jiao Tong University, Shanghai, China

**Keywords:** bilateral Wilms tumor, drug resistance, neoplasm metastasis, nephron-sparing surgery, TP53 mutations

## Abstract

Bilateral Wilms tumor (BWT) accounts for 5%–7% of all Wilms tumors. BWT presents with an increased complexity and poorer prognosis than unilateral tumors. Currently, the standard treatment involves neoadjuvant chemotherapy combined with nephron-sparing surgery to preserve renal function. While regimens established by leading cooperative groups have improved survival rates, patients with high-risk molecular alterations, such as TP53 mutations, often exhibit intrinsic chemoresistance, complicating their management. We report the case of a 3-year-old girl who presented with intermittent abdominal pain. Imaging demonstrated bilateral stage V WT, with multiple hepatic and pulmonary metastases and extensive lymphadenopathy. She was managed according to the Chinese Children Cancer Group (CCCG)-WT-2019 protocol, receiving neoadjuvant VAD (vincristine, actinomycin D, and doxorubicin) chemotherapy, followed by staged bilateral nephron-sparing surgery. Histopathological examination demonstrated a high-risk, blastemal-predominant WT. Molecular analysis identified a *TP53* p.R273H missense mutation. Fluorescence *in situ* hybridization (FISH) analysis further demonstrated 1q gain and 16q deletion. Postoperative treatments included targeted oral therapy, multiple chemotherapy regimens, and radiotherapy; however, the disease progressed. Ultimately, the tumor advanced to end-stage disease, and the patient died. This highlights the limitations of current histology- and stage-based approaches for specific molecular subtypes. Current evidence supports more aggressive surgical intervention for high-risk cases, but it is essential to balance the need for renal function preservation with the goal of achieving oncological control.

## 1 Introduction

Wilms tumor (WT), or nephroblastoma, is the most prevalent pediatric renal malignancy, comprising over 90% of all kidney tumors in children (1). While these tumors typically arise within the kidney, they can, in rare instances, develop in extra-renal sites, a condition referred to as extra-renal Wilms tumor (ERWT) (2). With the implementation of modern multidisciplinary treatment protocols, the overall prognosis is favorable, with long-term survival rates exceeding 90% in patients with low-risk diseases (3). However, bilateral Wilms tumor (BWT) constitutes a distinct clinical subgroup, accounting for approximately 5%–10% of all cases, and presents significant therapeutic challenges (4). Compared to their unilateral counterparts, children with BWT face a substantially elevated risk of developing end-stage renal disease, potentially necessitating renal replacement therapy, such as dialysis or transplantation (5). Consequently, a paramount therapeutic objective is the complete eradication of tumor tissue while preserving as much functional renal parenchyma as possible.

The current standard of care for BWT, established by the leading cooperative groups, including the Children's Oncology Group (COG), the International Society of Pediatric Oncology (SIOP), and the Chinese Children Cancer Group (CCCG), converges on a consensus strategy: a risk-adapted approach centered on neoadjuvant chemotherapy followed by nephron-sparing surgery (6). This strategy aims to reduce tumor volume and facilitate renal preservation. Despite these standardized protocols, a subset of high-risk patients continues to experience poor outcomes. This group is often characterized by the presence of metastatic disease at diagnosis or the development of resistance to conventional chemotherapy regimens. For these children, therapeutic options remain limited, and survival rates are significantly diminished.

Recent molecular profiling studies have begun to unravel the biological heterogeneity underlying WT and its correlation with clinical behavior. Specific genomic alterations, including *TP53* mutations, gain of 1q, and loss of heterozygosity at 16q, have been identified as independent prognostic biomarkers associated with unfavorable outcomes (5, 7, 8). LOH of 16q is generally considered clinically significant primarily when it occurs in conjunction with 1p LOH or the presence of positive lymph nodes (7). Notably, patients harboring germline *TP53* mutations not only exhibit hereditary cancer predisposition syndrome but also frequently develop tumors with heightened aggressiveness and intrinsic chemoresistance. Although contemporary clinical guidelines are increasingly incorporating these molecular markers, further evidence from real-world clinical cohorts is essential to precisely delineate their impact on disease progression and treatment response in BWT.

Here, we present a fatal case of therapy-resistant BWT in a child with a germline *TP53* mutation, coupled with 1q gain and 16q deletion, who presented with synchronous pulmonary metastases. Despite receiving guideline-concordant neoadjuvant chemotherapy and undergoing sequential surgical interventions, the patient experienced rapid disease progression with multiorgan dissemination to the liver, lung, and lymph nodes. The tumor was refractory to first-line chemotherapy [vincristine, actinomycin D and doxorubicin (VAD)] and salvage regimens (etoposide–carboplatin). Subsequent experimental therapies and targeted agents also failed to achieve disease control, highlighting the critical unmet need for novel, biologically informed treatment approaches for molecularly defined, high-risk BWT.

## 2 Case description

**Figure 1:**
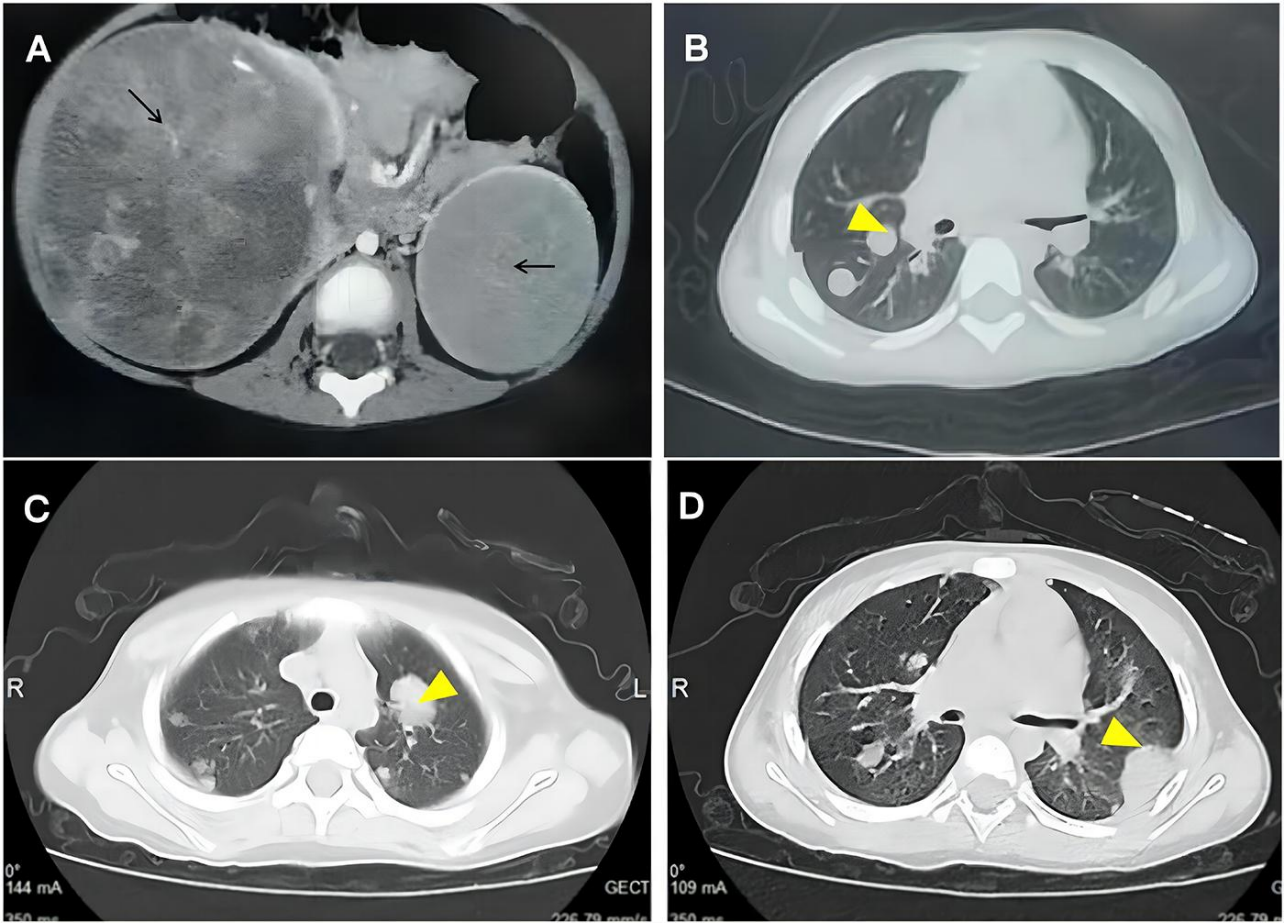
The patient's CT. **(A)** Right renal mass measuring approximately 9 × 8 × 10 cm, with multiple gravel-like calcifications and focal necrosis areas observed within. Post-contrast imaging shows significant heterogeneous enhancement. The left renal lesion measures approximately 5 × 6 × 6 cm, with focal necrosis observed, and shows uneven enhancement post-contrast; **(B)** Multiple metastatic tumors in both lungs; **(C)** Multiple, bilateral, round-shaped nodules were seen in both lungs, with a larger mass observed in the left upper lobe; **(D)** Multiple nodules and masses were seen in both lungs, with lesions in the right lower lobe and left lower lobe demonstrating irregular margins. Multiple linear opacities were present, suggesting possible interstitial changes.

A 3-year-old girl was initially admitted to our hospital with paroxysmal abdominal pain. Physical examination revealed a palpable mass on the right abdomen. Abdominal ultrasonography revealed large bilateral solid renal masses. Subsequently, the patient was transferred to a tertiary hospital, where contrast-enhanced computed tomography (CT) confirmed BWT with pulmonary metastases (Figures 1A,B). Head magnetic resonance imaging (MRI) showed no intracranial abnormalities. The patient was born macrosomic, and prenatal ultrasonography indicated mild bilateral renal enlargement with pyelectasis.

**Figure 2:**
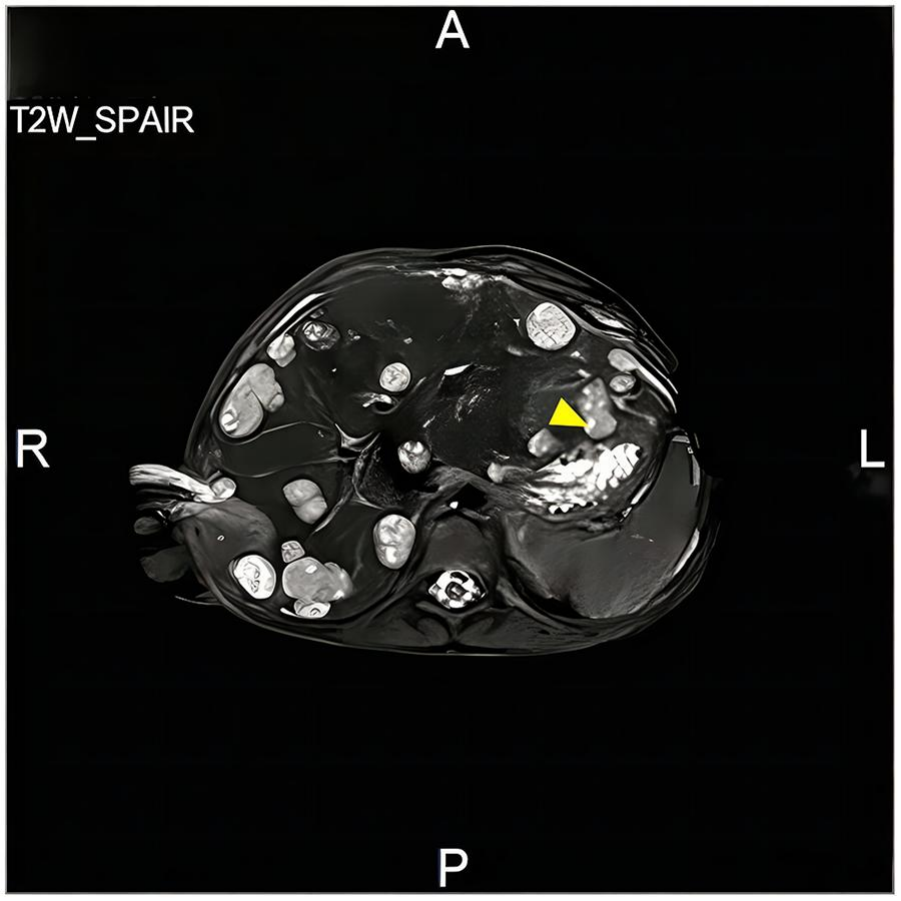
The patient's abdominal MRI, T2W_SPAIR sequence, demonstrates multiple hyperintense masses within the liver.

In accordance with the COG-WT-2019 protocol, the patient commenced preoperative chemotherapy with the VAD regimen, and completed two cycles before surgery. Following neoadjuvant therapy, imaging demonstrated that both tumors were localized to the lower poles of the kidneys, without extension into the renal pelvis, thereby rendering bilateral nephron-sparing surgery feasible. The patient first underwent left renal tumor resection with retroperitoneal lymph node dissection. During the procedure, the renal hilum was meticulously dissected, and renal pedicle clamping was employed during tumor resection. The tumor was resected through an incision placed at the interface between the tumor and the adjacent normal renal tissue, allowing complete excision along the tumor pseudocapsule. To preserve maximal renal parenchyma and reduce the risk of postoperative acute renal failure, the bilateral procedures were performed in a staged manner. Pathological examination demonstrated a high-risk blastemal-predominant nephroblastoma, with metastatic involvement of one to five lymph nodes. Postoperatively, she received further chemotherapy with cyclophosphamide and etoposide). Subsequently, patient underwent right renal tumor resection. Postoperative pathological assessment confirmed a chemotherapy-treated right-sided nephroblastoma with blastemal-type features, showing invasion into the perirenal adipose tissue without involvement of the surgical margin. Approximately 1-month post-surgery, re-evaluation at our facility demonstrated disease progression. Imaging studies revealed an increase in the residual right renal mass, along with new metastases to the lungs (Figures 1C,D), as well as the pleura, liver, and abdominal lymph nodes (Figure 2). Pathological examination confirmed a high-risk, blastemal-predominant WT. Fluorescence *in situ* hybridization (FISH) analysis demonstrated a 1q gain and 16q deletion. Next-generation sequencing (NGS) of the tumor sample identified a TP53 c.818G > A (p.Arg273His) mutation at a variant allele frequency (VAF) of 83.47% in a specimen containing 60% tumor cells, as assessed by HE staining. Germline Sanger sequencing performed on a buccal swab specimen confirmed the heterozygous mutation, establishing its germline origin.

**Figure 3:**
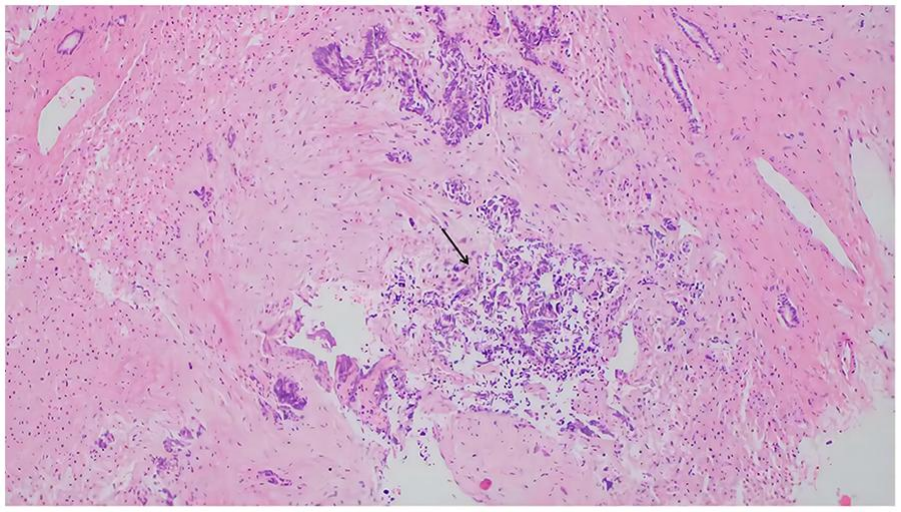
Pathological features of nephroblastoma, mixed epithelial and blastemal subtype. Hematoxylin–eosin staining reveals hyperchromatic, degenerated dysplastic cells, original magnifcation 10×.

Both tumors remained blastemal predominant following neoadjuvant chemotherapy, consistent with SIOP high-risk post-treatment histology and supporting the need of chemotherapy. Accordingly, second-line salvage chemotherapy was initiated, using etoposide and carboplatin; however, the patient's condition continued to worsen. Multiple subsequent regimens were attempted, including combinations of irinotecan with cyclophosphamide, as well as irinotecan with etoposide and temozolomide. A liver biopsy confirmed hepatic metastasis of the WT (Figure 3). Immunohistochemical staining revealed WT1 and CK positivity, a high Ki-67 proliferation index of 90%, and negative results for synaptophysin (SYN), PHOX2B, cyclin D1, *β*-catenin, PD-1, PD-L1, NKX2.2, SMA, and desmin. During the course of the disease, a tumor thrombus was identified within the right renal vasculature.

The patient subsequently underwent localized abdominal radiotherapy comprising seven sessions, each delivering 1.8 Gy. The tumor chemosensitivity gene testing predicted high sensitivity to several agents, including anthracyclines, etoposide, temozolomide, and cyclophosphamide, whereas reduced sensitivity was observed to platinum-based agents and taxanes. The predicted toxicity risk associated with irinotecan was moderate, which further informed the selection of subsequent chemotherapy regimens. To control disease progression, a combination regimen of targeted therapy with anlotinib and multi-agent chemotherapy (cyclophosphamide 440 mg/m2d1-d4 + etoposide100 mg/m2 d1-d4 and vincristine 1.5 mg/m2 d1 + irinotecan 50 mg/m2 d1-5 + temozolomide 150 mg/m2 d1-d5) was administered.

**Figure 4:**
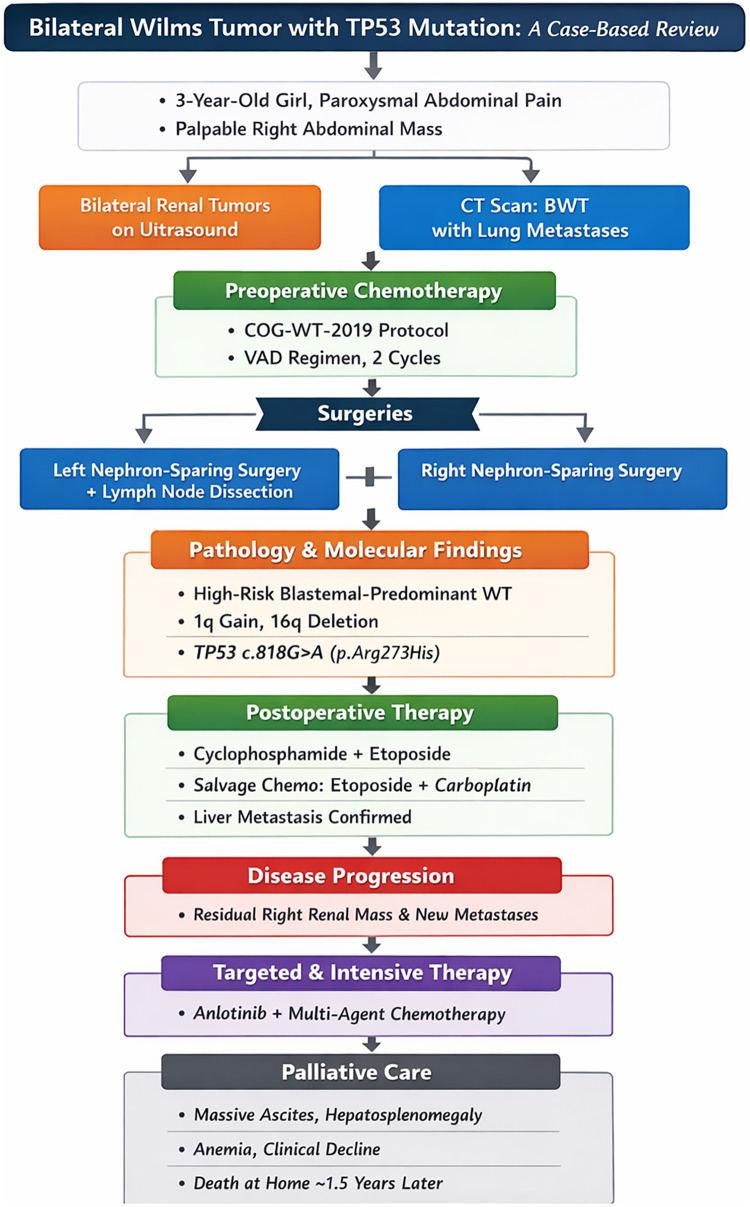
Diagnostic and treatment timeline of a 3-year-old girl with bilateral Wilms tumor harboring a germline TP53 mutation.

The patient exhibited terminal-stage features, including massive ascites, significant hepatosplenomegaly (with the liver edge palpable 15 cm below the costal margin), anemia, and a worsening clinical condition. Despite the continued therapy, disease control was not achieved. The family elected to discontinue antitumor treatment and transitioned to palliative care. No further interventions were performed after discharge, and the patient died at home approximately 1.5 years later. The diagnostic and treatment course of the patient is summarized in Figure 4.

## 3 Discussion

The management of BWT remains a formidable clinical challenge, requiring a careful balance between maximizing oncological cure and preserving long-term renal function (9, 10). Findings from the AREN0534 study suggest that tailoring the duration and intensity of chemotherapy according to postoperative histopathological response may improve survival outcomes in BWT. Although contemporary multimodal therapy has substantially improved overall survival, the present case illustrates the clinical course of a child with therapy-refractory BWT, *TP53* mutation, and multiple high-risk molecular markers, highlighting critical limitations in current treatment paradigms. This case further reinforces the concern that, for molecularly high-risk BWT driven by alterations, such as *TP53* mutations, the therapeutic efficacy of current standard chemotherapy regimens is profoundly limited, culminating in a very poor prognosis, even when management strictly adheres to established clinical guidelines (4).

The current treatment strategy centers on nephron-sparing surgery as the cornerstone of management, supported by evidence demonstrating superior long-term renal outcomes compared with radical nephrectomy (11, 12). In pediatric patients, loss of more than 50% of renal parenchyma is associated with an increased risk of renal insufficiency. Staged resection offers two key advantages: it avoids excessive loss of renal parenchyma during a single procedure and shortens the duration of renal pedicle clamping, thereby reducing the risk of postoperative acute renal injury.

Approximately 10% of pediatric cancer patients harbor genetic mutations predisposing them to cancer, with this percentage rising to 30% in cases of WT, including BWT. Certain susceptibility states are associated with specific histological and clinical features, suggesting differences in the mechanisms underlying tumorigenesis (13). The central role of *TP53* as a tumor suppressor is well-established, and germline pathogenic variants are classically associated with Li-Fraumeni syndrome (LFS). Its presence in WT predicts a drastically poor treatment response and survival outcomes (14). Germline *TP53* mutations, associated with LFS, are increasingly recognized as conferring susceptibility to WT. Analysis of 3034 patients in the International Agency for Research on Cancer *TP53* database demonstrated that germline *TP53* mutations significantly increase the risk of WT, particularly among patients fulfilling LFS criteria (15). A Dutch study conducted between 2015 and 2020 reported that 4% of 126 WT patients carried germline *TP53* mutations, and 4 of these tumors exhibited a second somatic “hit,” consistent with Knudson's two-hit hypothesis. *TP53* missense mutations, including p.R273H, has also been reported in association with bilateral WT (7, 16). *TP53* alterations are identified in approximately one-third of diffuse anaplastic WT cases and are frequently correlating with immune/stromal cell depletion and poor prognosis, suggesting a critical role in tumor progression (17). In WT with diffuse atypical hyperplasia, *TP53* mutations are found in virtually all classical atypical regions, accompanied by jump evolution and loss of wild-type alleles, indicating that germline *TP53* mutations may drive early clonal evolution (18). The 2023 American Cancer Society guidance recognizes WT as one of the malignancies associated with LFS and recommends lifetime surveillance for individuals with pathogenic *TP53* variants (19). In a study of 137 WT patients, 52% carried pathogenic germline or mosaic mutations, underscoring the genetic susceptibility, with *TP53* identified as a key gene (13). Further evidence has shown that 57% of cases have germline mutations, with *TP53* among the implicated genes (9). Although some studies do not directly address WT, current evidence supports that germline *TP53* mutations are a significant risk factor, especially in high-risk cases with features like diffuse anaplasia, early onset, or bilateral involvement. Li-Fraumeni syndrome patients have a lifetime cancer risk exceeding 90%, with WT being a common childhood solid tumor in this group (20). Taken together, germline *TP53* mutations lead to *p53* dysfunction, promote aberrant proliferation and differentiation of renal precursor cells, and, in conjunction with secondary somatic mutations, significantly increase WT risk, particularly in specific histological subtypes and clinical phenotypes (21). In addition, *TP53* mutations serve as prognostic indicators in treatment, potentially predicting poor chemotherapy response, because the genomic instability they promote can facilitate the evolution of drug-resistant clones. Research has found that *TP53* mutations often co-occur with *SIX1* and *MYCN* mutations in relapsed tumors and, in some cases, are associated with *CDKN2A* mutations or a higher tumor mutational burden (22). *TP53* mutations are associated with dysregulation of genes involved in the cell cycle and DNA repair, leading to increased genomic instability and a significant reduction in tumor cell sensitivity to DNA-damaging chemotherapeutic agents. The strong association between anaplastic histological features and *TP53* alterations may represent a key mechanism for the rapid metastasis and vascular invasion observed in this case (23).

Concurrently, 1q gain and 16q deletion have been recognized as robust independent prognostic markers of unfavorable outcomes. The 1q region harbors multiple oncogenes implicated in cell proliferation and survival, and 1q gain leads to increased copy number and consequent upregulation of these oncogenes, thereby significantly promoting malignant tumor progression (24). Conversely, pathogenic variants in ciliary-related genes located in the 16q region may be the underlying cause of the enhanced invasiveness observed in these tumors (25). The clinical significance of the vascular invasion observed in this case warrants careful consideration. The early postoperative development of liver metastases and renal hilar vascular tumor thrombus not only is consistent with the rapid disease dissemination but also supports an unusually aggressive biological behavior. From a molecular mechanism perspective, *CNOT2*, a gene involved in the TP53-associated co-expression network of the *TP53* signaling pathway, accelerates tumor cell proliferation and angiogenesis via VEGFR signal transduction (26). This mechanism may be associated with the enhanced vascular penetration and distant metastatic capacity observed in the tumor cells of our patient. Notably, despite the administration of anlotinib, a multitarget anti-angiogenic agent, it failed to control disease progression, suggesting that, in the context of *TP53* mutations, tumor cells may activate alternative signaling pathways to sustain their invasive potential.

Despite strict adherence to the CCCG-WT-2019 protocol throughout the treatment course—including neoadjuvant VAD chemotherapy, staged nephron-sparing surgeries, and subsequent standard salvage therapy with etoposide and carboplatin—the tumor exhibited explosive and widespread progression in the immediate postoperative period. This tragic outcome underscores the fundamental limitations of current histology- and clinical staging-based treatment paradigms when confronted with specific biologically aggressive molecular subtypes. Importantly, although a staged nephron-sparing approach was appropriately employed to preserve renal function (11, 12), the rapid progression of the disease indicates that even optimal surgical strategy may be insufficient to overcome the intrinsic biological aggressiveness driven by high-risk molecular features.

This treatment failure underscores several priorities for high-risk BWT with germline *TP53* mutations. First, precision-based therapeutic strategies directed at specific molecular alterations is essential. In *TP53*-mutant tumors, agents such as WEE1 and ATR inhibitors (27), as well as PI3 K/AKT/mTOR pathway inhibitors (28) represent promising strategies, with platforms like Pediatric MATCH facilitating access for refractory patients (29). Second, enhanced preoperative evaluation integrating routine genetic testing (e.g., *TP53*, WT1) with functional imaging could better predict chemotherapy sensitivity and surgical outcomes (30). Third, advanced diagnostic technologies such as liquid biopsy for circulating tumor DNA monitoring may enable earlier detection of minimal residual disease, particularly given the rapid postoperative progression observed in this case. Finally, for patients with multifocal metastases, interventional modalities like transarterial chemoembolization (TACE) and selective internal radiotherapy offer options for local disease control (31, 32).

In summary, we report a case of BWT with high-risk molecular features that progressed despite intensive multimodal therapy. This case highlights the critical challenge of balancing oncological control against preservation of renal function in patients with aggressive molecular subtypes. Current treatment protocols based predominantly on histology and stage may be insufficient for tumors harboring *TP53* mutations and other high-risk alterations. We recommend incorporating molecular profiling and targeted therapies into initial management to improve survival while optimizing quality of life for these young patients.

## Data Availability

The original contributions presented in the study are included in the article/Supplementary Material, further inquiries can be directed to the corresponding authors.
